# Association between high‐sensitivity C‐reactive protein, lipoprotein‐associated phospholipase A2 and carotid atherosclerosis: A cross‐sectional study

**DOI:** 10.1111/jcmm.13803

**Published:** 2018-08-09

**Authors:** Huamin Liu, Yan Yao, Youxin Wang, Long Ji, Kai Zhu, Haitao Hu, Jianxin Chen, Jichun Yang, Qinghua Cui, Bin Geng, Qing Liu, Dong Li, Yong Zhou

**Affiliations:** ^1^ School of Public Health Taishan Medical University Tai'an China; ^2^ Department of Cardiology Beijing Anzhen Hospital Capital Medical University Beijing China; ^3^ Beijing Key Laboratory of Clinical Epidemiology School of Public Health Capital Medical University Beijing China; ^4^ Department of Microbiology and Immunology University of Texas Medical Branch Galveston Texas; ^5^ Beijing University of Chinese Medicine Beijing China; ^6^ Department of Physiology and Pathophysiology School of Basic Medical Sciences Key Laboratory of Molecular Cardiovascular Science of the Ministry of Education Center for Non‐coding RNA Medicine Peking University Health Science Center Beijing China; ^7^ Department of Biomedical Informatics School of Basic Medical Sciences Key Laboratory of Molecular Cardiovascular Science of the Ministry of Education Center for Non‐coding RNA Medicine Peking University Health Science Center Beijing China; ^8^ Hypertension Center Fuwai Hospital State Key Laboratory of Cardiovascular Disease Chinese Academy of Medical Sciences and Peking Union Medical College Beijing China; ^9^ The Department of Neurosurgery Xiangya Hospital Central South University Changsha China; ^10^ Beijing Institute of Heart, Lung and Blood Vessel Diseases Beijing Anzhen Hospital Capital Medical University Beijing China

**Keywords:** carotid atherosclerosis, combination, high‐sensitivity C‐reactive protein, lipoprotein‐associated phospholipase A2

## Abstract

High‐sensitivity C‐reactive protein (hs‐CRP) and lipoprotein‐associated phospholipase A2 (Lp‐PLA2) have been reported to be independent predictors of atherosclerosis. However, whether the combination of these two markers can improve the prediction of atherosclerosis is unknown. This study aimed to evaluate the association between combining hs‐CRP and Lp‐PLA2 and predicting carotid atherosclerosis. A total of 1982 participants aged ≥40 years were included in this study. Hs‐CRP and Lp‐PLA2 were measured by a high‐sensitivity nephelometry assay and quantitative sandwich enzyme‐linked immunosorbent assay, respectively. Ultrasonography was performed on the bilateral carotid arteries to evaluate stenosis and plaques. Multivariable logistic regression models were used to analyse the association between the combination of the hs‐CRP and Lp‐PLA2 levels and carotid plaques and stenosis. A total of 1579 (79.7%) and 181 (9.1%) subjects had carotid plaques and carotid stenosis, respectively. The group with high hs‐CRP and Lp‐PLA2 levels had the highest prevalence of carotid plaques (90.6%) and stenosis (20.8%). A significant association was found between high hs‐CRP and Lp‐PLA2 levels and carotid stenosis (adjusted odds ratio [OR]: 2.39; 95% confidence interval [CI]: 1.13‐5.09), but this combination was not associated with carotid plaques (OR: 2.62, 95% CI: 0.93‐7.38). The results suggested that the combination of hs‐CRP and Lp‐PLA2 were better predictors than either protein alone with regard to carotid atherosclerosis.

## 1 INTRODUCTION

Carotid atherosclerosis is a primary cause of stroke and disability worldwide.^1^, ^2^ Inflammation is an important predictor of carotid atherosclerosis and plays a critical role in the formation and development of plaques and enhancement of the intima media thickness (IMT).^3^ Many inflammatory biomarkers, including lipoprotein‐associated phospholipase A2 (Lp‐PLA2), fibrinogen, serum amyloid A (SAA) and interleukin‐6 (IL‐6), have been approved by the United States Food and Drug Administration (FDA) as predictors of ischemic stroke; additionally, high‐sensitivity C‐reactive protein (hs‐CRP) has been associated with carotid atherosclerosis.^4^

Hs‐CRP is secreted by the liver in response to IL‐6 following a microbial trigger or tissue damage. Numerous studies have shown that elevated hs‐CRP levels are significantly associated with an increased risk of carotid atherosclerosis.^5^, ^6^ Indeed, adding CRP to the Framingham risk score improved the cardiovascular risk prediction.^7^ Nevertheless, a significant association of hs‐CRP with atherosclerosis has not been demonstrated in all epidemiological studies.^8^, ^9^ Compared to hs‐CRP, Lp‐PLA2 has the advantage of being a more specific marker for cardiovascular risk.^7^ However, epidemiological studies conducted in different populations have found inconsistent results regarding whether Lp‐PLA2 can be used as a predictor of atherosclerosis.^10^ This controversy has fuelled the search for more effective prediction methods for cardiovascular disease.

Several prospective studies have demonstrated that the combination of hs‐CRP and Lp‐PLA2 improves the prediction of the risk of cardiovascular diseases, including coronary artery disease and stroke.^7^, ^11^ However, the association of hs‐CRP with Lp‐PLA2 for carotid atherosclerosis is less understood. The aim of this study was to evaluate the association of the combination of hs‐CRP and Lp‐PLA2 with carotid atherosclerosis in participants with 40 years or older, who participated in the Asymptomatic Polyvascular Abnormalities Community (APAC) study. The carotid atherosclerosis risk prediction obtained by measuring hs‐CRP and Lp‐PLA2 was discussed.

## 2 METHODS

### 2.1 Study population

**Figure 1:**
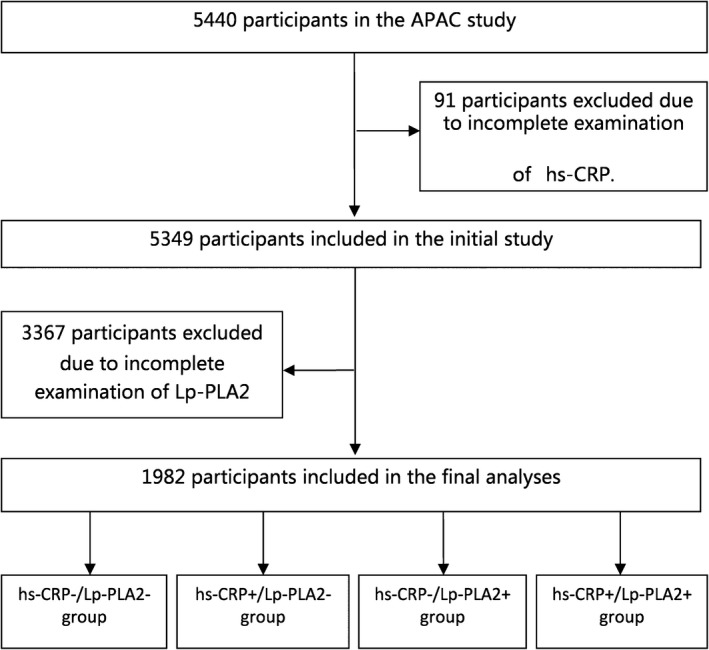
Flowchart of the study. APAC, Asymptomatic Polyvascular Abnormalities Community; hs‐CRP, highly sensitive C‐reactive protein; hs‐CRP−, low hs‐CRP; hs‐CRP+, high hs‐CRP; Lp‐PLA2, lipoprotein‐associated phospholipase A2; Lp‐PLA2−, low Lp‐PLA2; Lp‐PLA2+, high Lp‐PLA2

The APAC study is a community‐based, observational cohort study aimed at investigating the epidemiology of asymptomatic polyvascular abnormalities in Chinese adults.^12^, ^13^ The inclusion criteria of the APAC study are as follows: (a) ≥40 years old; (b) complete basic information available; and (c) no history of stroke, myocardial infarction, coronary heart disease, transient ischemic attack and neurological deficits.^14^, ^15^ Among the 5440 participants in the APAC study, 1982 participants (mean age 60 ± 11.7 years) with available hs‐CRP and Lp‐PLA2 levels were included in the final analyses (Figure 1). The present study was performed according to the guidelines of the Helsinki Declaration and was approved by the ethics committees of Kailuan General Hospital and Beijing Tiantan Hospital. Written informed consent was obtained from all participants.

### 2.2 Assessment of carotid stenosis and carotid plaque

Bilateral carotid duplex sonography (ACUSON X300, Siemens, Germany) was used to evaluate the presence of carotid stenosis and carotid plaques.^1^, ^12^ The investigators were blinded to the clinical and laboratory examination results of the study participants. A carotid plaque was defined as a focal structure either invading >0.5 mm into the arterial lumen, or invading into at least 50% of the surrounding IMT, or with a thickness at least 1.5 mm from the intimae‐lumen interface to the media adventitia interface. Carotid stenosis was assessed using established ultrasound criteria as follows^1^: (a) an internal carotid artery peak systolic velocity ≤125 cm/s was defined as <50% stenosis; (b) an internal carotid artery peak systolic velocity 125‐230 cm/s and a visible plaque was defined as 50%‐69% stenosis and (c) an internal carotid artery peak systolic velocity >230 cm/s or a markedly narrowed lumen was defined as >70% stenosis. The carotid ultrasound examination results were reviewed by two independent reviewers. Discrepancies were resolved by discussion between the experts or a third examination.

### 2.3 Determination of the CRP and Lp‐PLA2 levels

Hs‐CRP was measured with a high‐sensitivity nephelometry assay (Cias Latex CRP‐H, Kanto Chemical Co. Inc., Tokyo, Japan). The hs‐CRP concentrations were categorized into two groups as follows^16^: high level (hs‐CRP+, >3 mg/L) and low level (hs‐CRP−, <3 mg/L).

The plasma Lp‐PLA2 concentration was measured using a high‐sensitivity, quantitative sandwich enzyme‐linked immunosorbent assay (Quantikine ELISA, R&D Systems Inc. Minneapolis, MN, USA) and categorized into two groups as follows: high level (Lp‐PLA2+, >200 ng/mL) and low level (Lp‐PLA2−, ≤200 ng/mL).^17^

All participants were divided into the following four groups: low hs‐CPR/low Lp‐PLA2 (hs‐CRP−/Lp‐PLA2−), high hs‐CRP/low Lp‐PLA2 (hs‐CRP+/Lp‐PLA2−), low hs‐CRP/high Lp‐PLA2 (hs‐CRP−/Lp‐PLA2+) and high hs‐CRP/high Lp‐PLA2 (hs‐CRP+/Lp‐PLA2+).

### 2.4 Covariates

Questionnaires were used to collect information from the participants, including demographic variables, history of disease, lifestyle and drug history. Clinical characteristics and biochemical indicators were assessed at Kailuan General Hospital. The covariates included the education level, monthly per capita income, smoking, alcohol intake, blood pressure, fasting blood glucose, body mass index (BMI), cholesterol, triglycerides, high‐density lipoprotein cholesterol (HDL‐C) and low‐density lipoprotein cholesterol (LDL‐C). The assessment of covariates was described in a previous study.^2^

### 2.5 Statistical analysis

All continuous variables in our study were normally distributed. Continuous variables were presented as the mean with standard deviation and compared using one‐way ANOVA. Categorical variables were compared using the chi‐square test. Logistic regression was used to evaluate the association between hs‐CRP, Lp‐PLA2 and carotid plaques and stenosis by calculating odds ratios (ORs) or adjusted ORs with 95% confidence intervals (CIs). To examine effect modification by hs‐CRP and Lp‐PLA2, we used a post‐estimation Wald test in multivariable‐adjusted logistic model to get an omnibus *P value* for interaction of hs‐CRP and Lp‐PLA2 with carotid stenosis and plaques. The variables adjusted for were age, sex, BMI, education, income, smoking status, alcohol consumption, hypertension, hyperlipidemia and diabetes mellitus Figure 1.

All statistical tests were two‐sided, and the significance level was set as *P *<* *0.05. The statistical analyses were performed using SAS software, version 9.3 (SAS Institute Inc., Cary, NC, USA).

## 3 RESULTS

### 3.1 Participant characteristics

**Figure 2:**
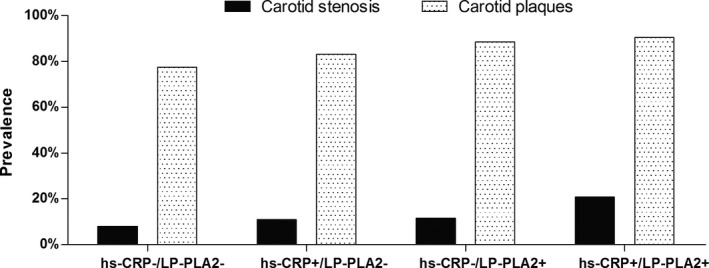
The proportion of participants with carotid plaques and carotid stenosis in the study groups. *hs‐CRP+, high hs‐CRP; hs‐CRP−, low hs‐CRP; Lp‐PLA2−, low Lp‐PLA2; Lp‐PLA2+, high Lp‐PLA2; High hs‐CRP was defined as ≥3 mg/L; high Lp‐PLA2 was defined as ≥200 ng/mL

Of the 5440 participants in the APAC study, 5349 and 2012 participants had recorded measurements for hs‐CRP and Lp‐PLA2, respectively. The prevalence of carotid plaques and carotid stenosis were 79.7% (1579/1982) and 9.1% (181/1982), respectively. The characteristics of the participants are summarized in Table 1. Significant differences were found among the groups in age, education level, income, BMI, smoking status, alcohol consumption, hypertension, diabetes, total cholesterol, triglycerides and HDL‐C (*P *<* *0.05). The prevalence of carotid plaques and carotid artery stenosis was higher in the hs‐CRP+/Lp‐PLA2+ group (90.6% and 20.8%, respectively, all *P *<* *0.05) than in the other three groups (Figure 2).

**Table 1:** Participant characteristics in the study groups

Characteristics	Total	hs‐CRP−/Lp‐PLA2−	hs‐CRP+/Lp‐PLA2−	hs‐CRP−/Lp‐PLA2+	hs‐CRP+/Lp‐PLA2+	*P*
Number of subjects (n, %)	1982	1428 (72.1)	354 (17.9)	147 (7.4)	53 (2. 7)	
Age (years)	60.3 ± 11.7	59.1 ± 11.1	61.6 ± 10.8	67.4 ± 13.7	67.0 ± 14.6	<0.001
Male (n, %)	1457 (73.5)	1060 (74.2)	257 (72.6)	107(72.8)	33(62.3)	0.260
Education level (n, %)						
Illiteracy/primary school	362 (18.3)	223 (15.6)	89 (25.1)	36 (24.5)	14 (26.4)	<0.001
Middle school	884 (44.6)	655 (45.9)	146 (41.2)	61 (41.5)	22 (41.5)	
College/university	736 (37.1)	550 (38.5)	119 (33.6)	50 (34.0)	17 (32.1)	
Income, ¥/month (n, %)						
≤¥1000	347 (17.5)	249 (17.4)	67 (18.9)	23 (15.8)	8 (15.1)	0.002
¥1000‐3000	1319 (66.6)	962 (67.4)	242 (68.4)	84 (57.5)	31 (58.5)	
≥¥3001	315 (15.9)	217 (15.2)	45 (12.7)	39 (26.7)	14 (26.4)	
Current smoker (n, %)	753 (38.0)	554 (38.8)	142 (40.1)	45 (30.6)	12 (22.6)	0.021
Current alcohol consumption (n, %)	317 (16.0)	251 (17.6)	0 (14.1)	13 (8.8)	3 (5.7)	0.004
Hypertension (n, %)	1149 (58.0)	796 (55.7)	228 (64.4)	92 (62.6)	33 (62.3)	0.014
Dyslipidemia (n, %)	1051 (53.0)	728 (51.0)	229 (64.7)	68 (46.3)	26 (49.1)	<0.001
Diabetes (n, %)	322 (16.2)	212 (14.8)	79 (22.3)	21 (14.3)	10 (18.9)	0.006
BMI (kg/m^2^)	24.9 ± 3.2	24.7 ± 3.1	26.1 ± 3.4	24.2 ± 3.1	24.6 ± 3.6	<0.001
TC (mmol/L)	5.2 ± 1.1	5.2 ± 1.1	5.3 ± 1.1	5.1 ± 1.2	5.1 ± 1.2	0.020
Triglycerides (mmol/L)	1.7 ± 1.4	1.6 ± 1.4	1.9 ± 1.6	1.5 ± 1.9	1.5 ± 0.8	0.002
HDL‐C (mmol/L)	1.6 ± 0.5	1.6 ± 0.5	1.5 ± 0.4	1.6 ± 0.5	1.5 ± 0.4	<0.001
LDL‐C (mmol/L)	2.7 ± 0.8	2.7 ± 0.8	2.6 ± 1.0	2.6 ± 0.7	2.6 ± 0.7	0.281

High hs‐CRP was defined as ≥3 mg/L; high Lp‐PLA2 was defined as ≥200 ng/mL.

BMI, body mass index; HDL‐C, high‐density lipoprotein cholesterol; hs‐CRP−, low hs‐CRP; hs‐CRP+, high hs‐CRP; LDL‐C, low‐density lipoprotein cholesterol; Lp‐PLA2−, low Lp‐PLA2; Lp‐PLA2+, high Lp‐PLA2; TC, total cholesterol.

### 3.2 Association between hs‐CRP, Lp‐PLA2 and carotid plaques and carotid stenosis

**Figure 3:**
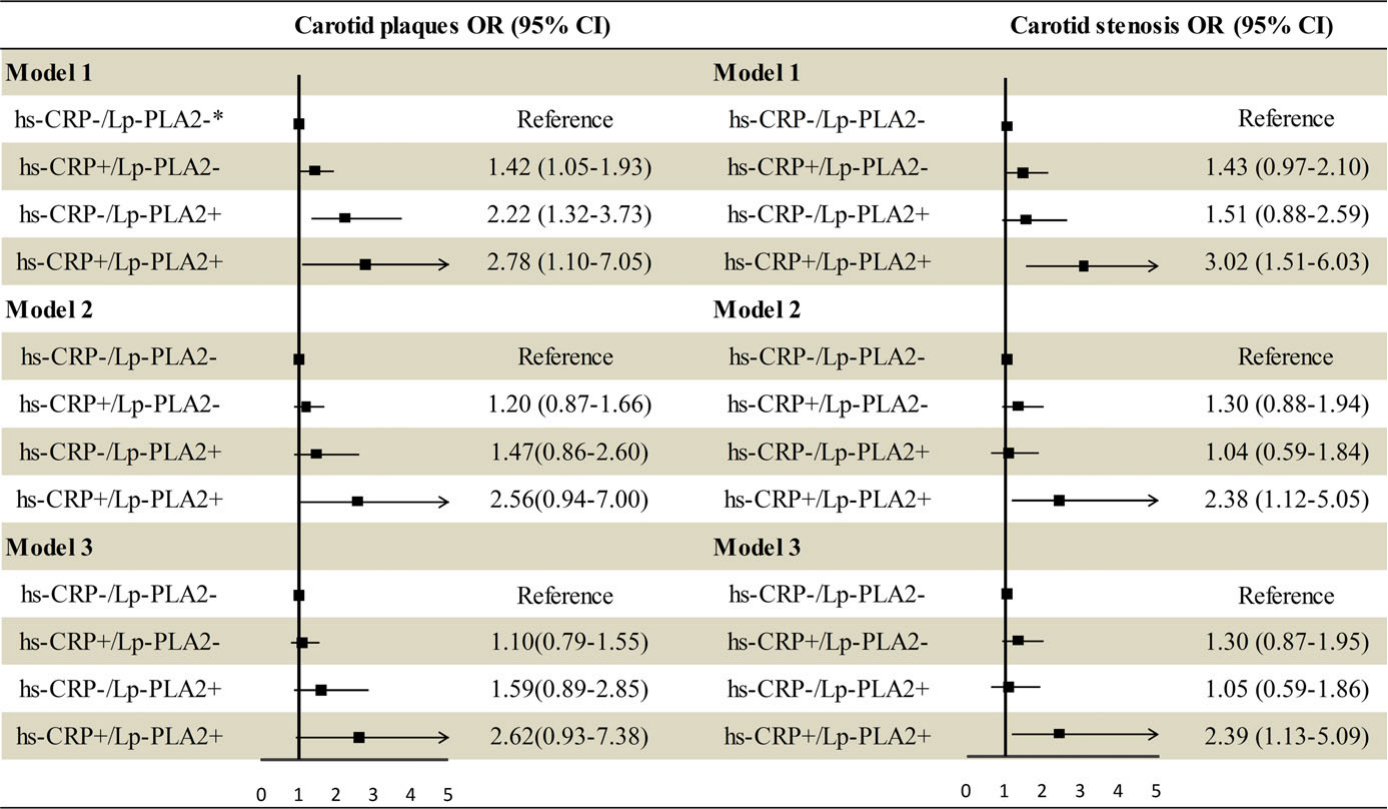
Association of hs‐CRP combined with Lp‐PLA2 and carotid plaques and carotid stenosis. *hs‐CRP+, high hs‐CRP; hs‐CRP−, low hs‐CRP; Lp‐PLA2−, low Lp‐PLA2; Lp‐PLA2+, high Lp‐PLA2; High hs‐CRP was defined as a CRP concentration ≥3 mg/L, High Lp‐PLA2 was defined as a Lp‐PLA2 concentration ≥200 ng/mL; Model 1: unadjusted; Model 2: adjusted for age, sex, education, income, smoking, and alcohol consumption; Model 3: adjusted for model 2 plus hypertension, hyperlipidemia, diabetes mellitus, and BMI

The associations between hs‐CRP combined with Lp‐PLA2 for carotid plaques and carotid stenosis are shown in Figure 3. ORs with 95% CIs for both the hs‐CRP and Lp‐PLA2 groups with carotid artery stenosis compared with those in the hs‐CRP−/Lp‐PLA2− group were as follows: 3.02 (1.51‐6.03) in Model 1, 2.38 (1.12‐5.05) in Model 2 and 2.39 (1.13‐5.09) in Model 3. The hs‐CRP+/Lp‐PLA2+ group had the highest OR of 2.39 (95% CI: 1.13‐5.09, *P value* for interaction: 0.256) compared with the hs‐CRP+/Lp‐PLA2− group with an OR of 1.30 (95% CI: 0.87‐1.95) and the hs‐CRP−/Lp‐PLA2+ group with an OR of 1.05 (95% CI: 0.59‐1.86) in the fully adjusted model.

A significant association was found between high levels of both hs‐CRP and Lp‐PLA2 with carotid plaques in the crude model (OR: 2.78, 95% CI: 1.10‐7.05, Model 1). In model 2, high levels of both hs‐CRP and Lp‐PLA2 were not significantly associated with carotid plaques (OR: 2.56, 95% CI: 0.94‐7.00), and this association remained non‐significant in model 3 (OR: 2.62, 95% CI: 0.93‐7.38).

## 4 DISCUSSION

In this community‐based study, the main finding was that the combination of hs‐CRP and Lp‐PLA2 was associated with the carotid stenosis risk. The results showed an additive effect of hs‐CRP combined with Lp‐PLA2 on risk prediction, which suggested a clinically relevant role for hs‐CRP combined with Lp‐PLA2 in carotid atherosclerosis.

Several studies have reported that elevated hs‐CRP and Lp‐PLA2 concentrations are significantly associated with atherosclerosis characterized by artery plaques and artery stenosis.^18^, ^19^ Significant associations have also been found between CRP combined with Lp‐PLA2 and atherosclerotic events, such as coronary heart disease and stroke.^7^, ^20^ An elevated Lp‐PLA2 level was shown to be significantly associated with carotid artery plaques, but this association became non‐significant when Mendelian randomization methods were adopted.^21^

Our findings provide evidence that the combination of the hs‐CRP and Lp‐PLA2 levels is sufficiently stable to predict carotid artery stenosis independent of other atherosclerotic risk factors. The significant association between high hs‐CRP and Lp‐PLA2 levels and carotid artery stenosis was still present even after the multivariable adjustments, demonstrating that hs‐CRP combined with Lp‐PLA2 is an independent risk factor for carotid artery stenosis. The elevated ORs in the hs‐CRP+/Lp‐PLA2+ group compared with the hs‐CRP+/Lp‐PLA2− and hs‐CRP−/Lp‐PLA2+ groups indicate a significant association between hs‐CRP and Lp‐PLA2 in combination and carotid artery stenosis. Moreover, the risk of carotid stenosis was increased 1.39 times in the hs‐CRP+/Lp‐PLA2+ group participants compared with the hs‐CRP−/Lp‐PLA2− group participants, indicating a synergistic effect of hs‐CRP and Lp‐PLA2 in atherosclerosis. Lp‐PLA2 is an enzyme that can hydrolyse oxidized phospholipids to generate oxidized fatty acids and lysophosphatidylcholine (lyso‐PC), both of which have proinflammatory properties. Oxidized fatty acids and lyso‐PC increase adhesion molecule expression. High hs‐CRP levels may also increase adhesion molecule and chemokine expression to promote vascular inflammation. Thus, high levels of both hs‐CRP and Lp‐PLA2 are a better maker of the atherosclerosis risk than a high level of either protein alone.^22^ However, we were unable to demonstrate that high levels of both hs‐CRP and Lp‐PLA2 were associated with carotid plaques, independent of traditional risk factors.^23^, ^24^ The high prevalence of carotid plaques may have led to a decrease in the statistical power.

Our study has several limitations. First, we measured the Lp‐PLA2 concentration but not the Lp‐PLA2 activity in the 2012 participants, which might be insufficient to evaluate the effect of Lp‐PLA2. Second, the study was performed based on the Chinese participants who were 40 years of age or older, so it may be not generalizable to other races or the young. Finally, a causal inference may not be drawn in this cross‐sectional study.

In conclusion, our results indicated that the combination of CRP and Lp‐PLA2 was associated with carotid stenosis. Our findings suggested that the combination of the CRP and Lp‐PLA2 levels might be a useful measure for vascular risk assessment and could be a potential therapeutic target for carotid atherosclerosis.

## CONFLICT OF INTEREST

The authors confirm that there are no conflicts of interest.
